# Development and test–retest reliability of a screening tool for axial spondyloarthritis

**DOI:** 10.1371/journal.pone.0269494

**Published:** 2022-07-08

**Authors:** Divya Shridharmurthy, Kate L. Lapane, Sara Khan, Esther Yi, Jonggyu Baek, Jonathan Kay, Shao-Hsien Liu

**Affiliations:** 1 Division of Epidemiology, Department of Population and Quantitative Health Sciences, T.H. Chan School of Medicine at UMass Chan Medical School, Worcester, Massachusetts, United States of America; 2 Clinical and Population Health Research Program, Morningside Graduate School of Biomedical Sciences at UMass Chan Medical School, Worcester, Massachusetts, United States of America; 3 Novartis Pharmaceuticals Corporation, East Hanover, New Jersey, United States of America; 4 Division of Rheumatology, Department of Medicine, T.H. Chan School of Medicine at UMass Chan Medical School, Worcester, Massachusetts, United States of America; 5 Division of Rheumatology, Department of Medicine, UMass Memorial Medical Center, Worcester, Massachusetts, United States of America; National Institute of Child Health and Human Development (NICHD), NIH, UNITED STATES

## Abstract

**Background:**

People with axial Spondyloarthritis (axSpA) suffer from lengthy diagnostic delays of ~7 years. The usage of screening tools to identify axSpA patients in primary care can reduce diagnostic delays by facilitating early referral to rheumatologic care. The purpose of this study was to examine the psychometric properties of a potential screening tool for patients with axSpA.

**Method:**

Content validity was evaluated by soliciting feedback from 7 rheumatologists regarding the relevance and content representativeness of the proposed screening questions. For the test-retest study, participants ≥18 years of age with chronic back pain (≥3 months) without a diagnosis of mechanical or inflammatory back pain (n = 91) were e-recruited through ResearchMatch. Participation included completing identical baseline and follow-up questionnaires ~14 days apart. Weighted quadratic kappa was used to measure test-retest reliability between the two ratings of the ordinal scales. Construct validity was examined using exploratory factor analysis (EFA) and items with factor loadings ≥0.6 were extracted. Scale dimensionality and simplified factorial solutions were measured using Kaiser’s criteria (Eigenvalue >1). Cronbach’s alpha was used to measure internal consistency.

**Results:**

Most participants were women, non-Hispanic white, and had at least some college education, with a mean age of 45 years. On average, the age at onset of back pain was 31 years. Eleven questions yielded test–retest reliabilities ranging from 0.6 to 0.76. Results from EFA extracted two factors relating to: 1) how pain affects daily life functioning and 2) whether pain improves with movement. Internal consistency was high for questions evaluating how pain affects life, with a Cronbach’s alpha of 0.81. Following assessment for validity and reliability, the questionnaire was revised to create the 6-item screening tool.

**Conclusions:**

The 6-item SpA-SED screening tool designed to identify potential cases of axSpA was found to have good test–retest reliability and high internal consistency.

## Introduction

Axial spondyloarthritis (axSpA), is characterized by chronic back pain and stiffness, limited axial skeletal mobility, and fatigue [1,2]. The average delay in diagnosing an individual with axSpA ranges from 7 to 10 years [3] with estimates as high as 13 years between symptom onset and diagnosis in the United States [4]. Reasons for delay in disease diagnosis include: 1) common and vague initial symptoms (e.g., back pain) that are non-specific and could be ascribed to other conditions [5,6], and 2) appearance of radiological changes later in the disease course of axSpA [7,8]. Delayed diagnosis deprives patients of the potential benefit of early treatment in slowing disease progression to avoid or delay serious disability [9,10].

Delays in diagnosis have also been attributed to late referral of patients by general practitioners to rheumatologists, since non-rheumatologist physicians in the US are less aware of axSpA [11,12]. Studies show that primary care physicians have difficulty differentiating inflammatory back pain (IBP) from the more common mechanical back pain [13,14] or are unaware of other features of spondyloarthritis that are important for differential diagnosis [12,14]. We previously conducted qualitative research with primary care providers in which they agreed that improvements in screening and early detection of axSpA are needed [15]. The Assessment of SpondyloArthritis International Society (ASAS) Inflammatory Back Pain Assessment: ASAS Expert Criteria screening questions have been used in primary care settings to screen for axSpA [15,16]. However, primary care providers considered that some of these questions were neither sensitive nor specific and needed improvement [15,16].

Previously, we derived potential screening questions from qualitative interviews that we conducted with patients who had chronic back pain and refined the wording of these questions to improve their clarity and ease of administration as screening questions to be implemented in primary care settings [17]. The purpose of this study was to evaluate the psychometric properties of these proposed screening questions to develop a prognostic screening tool for axSpA for implementation in primary care settings.

## Methods

This study protocol of the SpondyloArthritis Screening and Early Detection (SpA-SED) Study–Test-retest study was approved by the UMass Chan Medical School Institutional Review Board (IRB number: H00020620).

### Study design

The study was conducted in two phases. First, a test-retest study was conducted among patients with chronic back pain, but who did not have a clinical diagnosis of either mechanical or inflammatory back pain. Participants were asked to complete identical baseline and follow-up questionnaires approximately 14 days apart. These questionnaires included 19 potential items/questions for inclusion in the screening tool (S1 and S2 Files). Questionnaires were administered online via REDCap [18], with the link to the follow-up questionnaire being sent approximately 14 days after the baseline questionnaire had been completed.

Second, a content validity study was conducted among members of a panel of rheumatologists with expertise in axSpA using REDCap to solicit feedback about the content validity of the proposed screening questions. Each rheumatologist was asked to specify his or her level of agreement with the relevance and content representativeness of the proposed items using a 5-point Likert scale (1 = disagree to 5 = strongly agree) (S3 File) [19]. The knowledge gained complemented and helped to interpret the data collected from patients in the test-retest study.

### Participant recruitment

Two groups of participants were recruited for the study: 1) patients with chronic back pain (≥3 months); and 2) rheumatologists with expertise in axSpA.

#### Patients

To be eligible for participation in on-line REDCap surveys, participants were required to: 1) have had back pain for at least 3 months (by self-report), but not have been given a clinical diagnosis of mechanical or inflammatory back pain; 2) have registered in ResearchMatch [20]; and 3) be at least 18 years of age. Patients were e-recruited through ResearchMatch, which is a disease-neutral, web-based recruitment registry to help match individuals who wish to participate in clinical research studies with researchers actively searching for volunteers throughout the United States [20].

Standard ResearchMatch procedures were followed to invite subjects to participate in our study. After receiving IRB approval, the study was registered on ResearchMatch and study details were entered. After IRB approval was verified by ResearchMatch, the research team was granted permission to access the database of potential study subjects. Individuals in the database who satisfied our eligibility criteria were identified. ResearchMatch then sent an IRB-approved recruitment message to these volunteers to inform them of the opportunity to participate in this study. After individuals had responded, giving permission to be contacted for our study, personal contact information was made available to the research team within the secure ResearchMatch system and these potential study participants were contacted on ResearchMatch. Patients who completed both the baseline and follow-up questionnaires were compensated for their participation with a $50 cash card.

**Fig 1:**
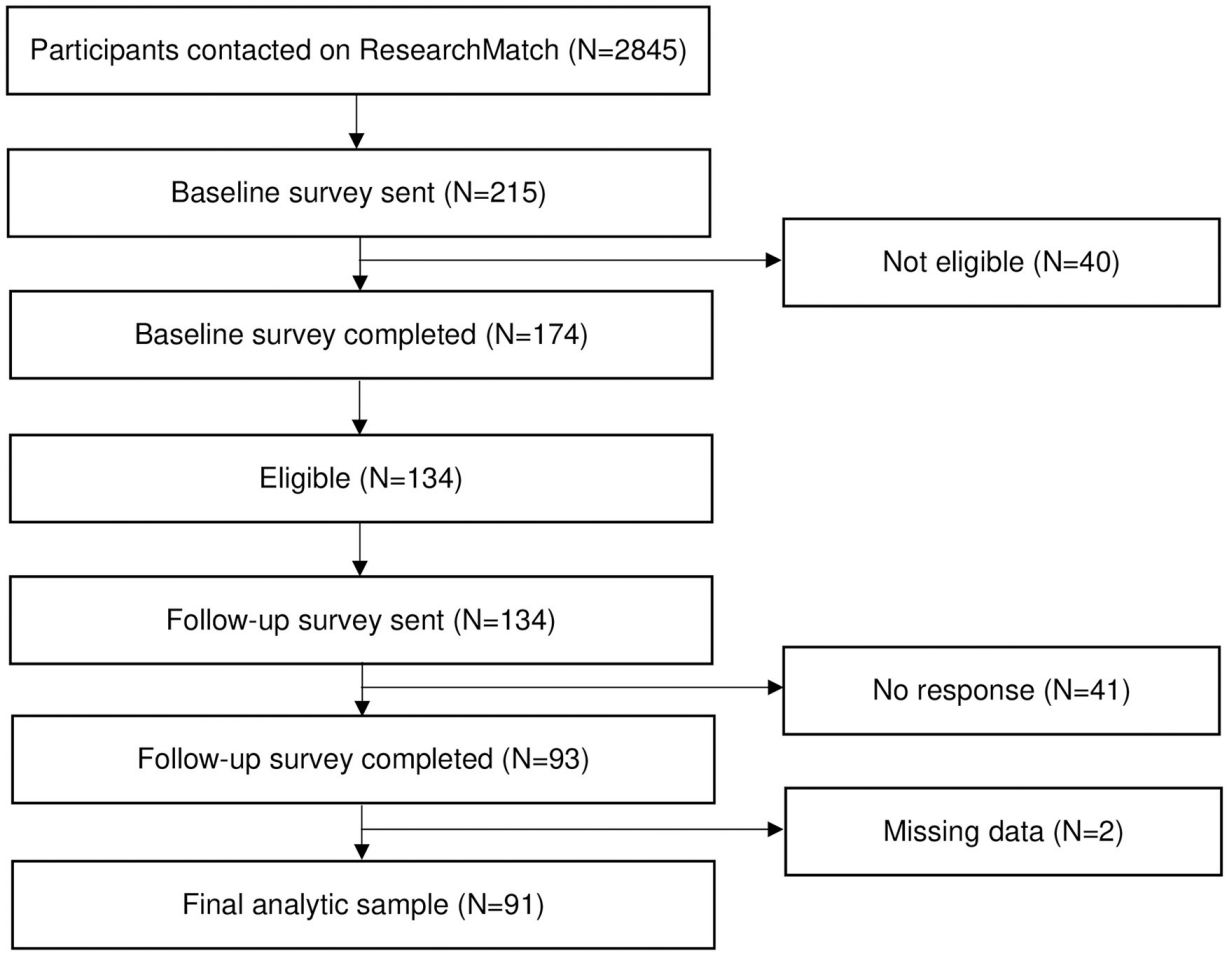
Flow chart of the recruitment process for study participants.

Fig 1 displays the patient recruitment flow chart. We contacted 2,845 potentially eligible participants on ResearchMatch. Of those, 215 individuals agreed to participate, and 174 completed the baseline questionnaire. Of these 174 subjects, 40 did not meet eligibility because of the absence of chronic back pain. Among the eligible 134 participants who completed the baseline questionnaire, all were sent the follow-up questionnaire and 93 completed the follow-up questionnaire. Ultimately, 91 participants responded to both the baseline and follow-up questionnaires with complete data.

#### Rheumatologists

Seven rheumatologists were identified from among members of the Spondyloarthritis Research and Treatment Network (SPARTAN) who were known by the investigators to have expertise in axSpA and were invited to participate in the study. Written informed consent was obtained. Those rheumatologists participating in the research study were compensated for their time and effort with a $300 cash card.

### Questionnaires

Each participant with chronic back pain completed an eligibility questionnaire at the beginning of the baseline survey (S1 File). The eligibility questionnaire confirmed whether the participant had suffered from back pain for ≥3 months and whether the back pain resulted from a specific incident. A background questionnaire, which collected information on patient demographics including sex/gender, age, race/ethnicity, and education level, was completed by eligible participants at the end of the baseline survey. Subjects were also asked if they would be willing to be re-contacted for future research.

### Statistical analysis

For patient participants, descriptive statistics including means and standard deviations (SD) for continuous variables and percentages for categorical variables were calculated to describe patient characteristics. Psychometric properties of the proposed screening tool were examined including test-retest reliability, exploratory factor analysis, and Cronbach’s alpha. Test-retest reliability was calculated by computing the percent agreement and k statistics between two administrations of the questionnaire to the same subject.

Overall, the analytic approach was an iterative process. First, weighted kappa was used to measure the test-retest reliability (i.e., agreement) between the two ratings of the ordinal scales [21,22]. With ordinal scales, weighted kappa coefficients are used where disagreements are weighted by the degree of discrepancy [21,23]. Quadratic weighted kappa is often recommended since it is equivalent to the product-moment correlation and the intraclass correlation coefficient under certain scenarios [22,24]. This metric typically varies from 0 (random agreement) to 1 (complete agreement). The kappa value of 0.6 was considered moderate test-retest reliability and was used as a cut-off to determine the selection of the proposed questions into the screening tool [25]. For questions with binary responses, the reliability was assessed using the Fleiss kappa coefficient [26,27].

Exploratory factor analysis (EFA) was then conducted to examine the dimensionality of the screening tool. Questions that did not have sufficient factor loading (e.g., a corrected item total correlation (CITC) > 0.3) was indicative of good fit and were included in the analysis [28]. Considering that our primary goal was to distill down the number of items needed to capture the underlying structure, only items with factor loadings ≥ 0.6 were extracted (factor loadings ≥ 0.5 suggest practical significance) [29]. The criterion used to produce scale dimensionality and simplified factorial solutions included Kaiser’s criteria (eigenvalue > 1 rule) [30] and the Scree plot [31].

Lastly, Cronbach’s alpha was used to measure internal consistency (i.e., scale reliability) of the screening tool, and to evaluate how closely the proposed screening questions were related [32]. Cronbach’s alpha is computed by correlating the score for each scale item with the total score for each observation (usually individual survey respondents or test takers), and then comparing that to the variance for all individual item scores. The resulting α coefficient of reliability ranges from 0 to 1 in providing this overall assessment of a measure’s reliability. A higher α coefficient indicates that more items have shared covariance and measure the same underlying concept [33].

For the questionnaire used to assess content validity by rheumatologists, the overall percent agreement for these items were evaluated and summarized. The rheumatologists were asked to rank each question based on its significance and relevance to axSpA symptoms using a 4-point Likert scale. Options ranged from being not important to very important. Agreement on each question was based on the option that received the highest level of agreement (majority of rheumatologists agreeing that the question is somewhat important) with incorporation of feedback from rheumatologists.

## Results

### Patient characteristics

Table 1 shows the demographic characteristic of patient participants. Overall, the majority of participants were women (72.5%), and non-Hispanic white (79%), with a mean age of 45 years. On average, the age at onset of back pain was 30.7 years. Although 31% of all subjects had completed college, 42% of men but only 29% of women had some college or a technical school degree.

**Table 1:** Sample characteristic of study participants.

Characteristics	Overall (n = 91)	Women (n = 66)	Men (n = 19)	Non-binary (n = 5)
Age, Mean (SD)	44.9 (15.2)	46.2 (15.2)	44.5 (14.8)	29.4 (8.3)
Age when first experienced back pain, Mean (SD)	30.7 (13.6)	31.6 (13.8)	31.5 (12.6)	16.0 (6.3)
Race, %				
Black	6.6	6.1	10.5	0
White	79.1	84.9	68.4	60.0
Latino	7.7	4.6	15.8	20.0
Asian	1.1	0	5.3	0
Hawaiian/ Other Pacific Islander	0	0	0	0
Native American Indian/ Alaskan Native	0	0	0	0
Multiracial	4.4	4.6	0	20.0
Education, %				
Less than high school	0	0	0	0
Completed high school or GED	7.7	10.6	0	0
Some college or technical school	33.0	28.8	42.1	60.0
Completed college	30.8	30.3	31.6	40.0
Graduate school	26.4	28.8	26.3	0
Other	1.1	1.5	0	0

### Test-retest reliability

The test-retest reliability for each proposed question is shown in Table 2. Eleven of the 19 items on the screener had relatively moderate correlations (quadratic kappa ≥ 0.6) across the two-week, test-retest period (Table 3). Those 11 questions yielded test–retest reliabilities with quadratic weighted kappa’s ranging from 0.60 to 0.76. Eight proposed questions with a quadratic kappa less than 0.60 were excluded from the screener (Table 4).

**Table 2:** Test-retest reliability of each proposed question.

	Quadratic weighted kappa	95% confidence interval	
Q1. Over the last 3 months, how often have you suffered from back pain without a known cause?	0.60	0.40	0.80
Q2. Over the last 3 months, how often did your back feel the same or worse?	0.56	0.35	0.76
Q3. Over the last 3 months, how often did your back feel stiff during the first two hours after waking?	0.75	0.55	0.96
Q4. Over the last 3 months, how often have you experienced a decreased range of motion?	0.62	0.42	0.83
Q5. Over the last 3 months, how often did your back pain get better with movement?	0.63	0.43	0.84
Q6. Over the last 3 months, how often did your back pain get better with movement after waking?	0.54	0.34	0.75
Q7. Over the last 3 months, how often did your back pain get better with movement within two hours after waking?	0.62	0.41	0.83
Q8. Over the last 3 months, how often have you been unable to sit still for more than two hours because of your back pain?	0.47	0.26	0.68
Q9. Over the last 3 months, how often did your back pain make it uncomfortable to sit for more than 2 hours?	0.73	0.52	0.93
Q10. Over the last 3 months, how often are you aware of your back pain when sitting for two hours or more?	0.51	0.30	0.71
Q11. Over the last 3 months, how often do you avoid social activities because of your back pain?	0.73	0.52	0.94
Q12. Over the last 3 months, how often did your back pain ease with rest?	0.46	0.26	0.66
Q13. Over the last 3 months, how often did your back pain make it difficult to sleep?	0.72	0.51	0.92
Q14. Over the last 3 months, how often did your back pain wake you up from sleep?	0.76	0.55	0.97
Q15. Over the last 3 months, how often did you have pain in your hip, neck, heel, or elbow?	0.63	0.43	0.84
Q16. Over the last 3 months, how often have you experienced alternating pain in your hips or buttocks?	0.58	0.37	0.79
Q17. Over the last 3 months, how often have you experienced improvement in your back pain after taking non-steroidal anti-inflammatory medications (like naproxen, ibuprofen, etc.)?	0.52	0.30	0.73
Q18*. Has a doctor ever told you that you have an autoimmune disease like iritis, Crohn’s disease, or psoriasis?	0.51	0.30	0.71
Q19*. Has anyone in your family had an auto immune disease?	0.70	0.49	0.91

*Q18 and Q19 were binary response.

**Table 3:** List of questions with moderate correlations (kappa ≥ 0.6).

	Quadratic weighted kappa	95% confidence interval	
Q1. Over the last 3 months, how often have you suffered from back pain without a known cause?	0.60	0.40	0.80
Q3. Over the last 3 months, how often did your back feel stiff during the first two hours after waking?	0.75	0.55	0.96
Q4. Over the last 3 months, how often have you experienced a decreased range of motion?	0.62	0.42	0.83
Q5. Over the last 3 months, how often did your back pain get better with movement?	0.63	0.43	0.84
Q7. Over the last 3 months, how often did your back pain get better with movement within two hours after waking?	0.62	0.41	0.83
Q9. Over the last 3 months, how often did your back pain make it uncomfortable to sit for more than 2 hours?	0.73	0.52	0.93
Q11. Over the last 3 months, how often do you avoid social activities because of your back pain?	0.73	0.52	0.94
Q13. Over the last 3 months, how often did your back pain make it difficult to sleep?	0.72	0.51	0.92
Q14. Over the last 3 months, how often did your back pain wake you up from sleep?	0.76	0.55	0.97
Q15. Over the last 3 months, how often did you have pain in your hip, neck, heel, or elbow?	0.63	0.43	0.84
Q19*. Has anyone in your family had an auto immune disease?	0.70	0.49	0.91

*Q19 was binary response.

**Table 4:** List of questions excluded (kappa < 0.6).

	Quadratic weighted kappa	95% confidence interval	
Q2. Over the last 3 months, how often did your back feel the same or worse?	0.56	0.35	0.76
Q6. Over the last 3 months, how often did your back pain get better with movement after waking?	0.54	0.34	0.75
Q8. Over the last 3 months, how often have you been unable to sit still for more than two hours because of your back pain?	0.47	0.26	0.68
Q10. Over the last 3 months, how often are you aware of your back pain when sitting for two hours or more?	0.51	0.30	0.71
Q12. Over the last 3 months, how often did your back-pain ease with rest?	0.46	0.26	0.66
Q16. Over the last 3 months, how often have you experienced alternating pain in your hips or buttocks?	0.58	0.37	0.79
Q17. Over the last 3 months, how often have you experienced improvement in your back pain after taking non-steroidal anti-inflammatory medications (like naproxen, ibuprofen, etc.)?	0.52	0.30	0.73
Q18*. Has a doctor ever told you that you have an autoimmune disease like iritis, Crohn’s disease, or psoriasis?	0.51	0.30	0.71

*Q18 was binary response.

### Exploratory factor analysis

**Fig 2:**
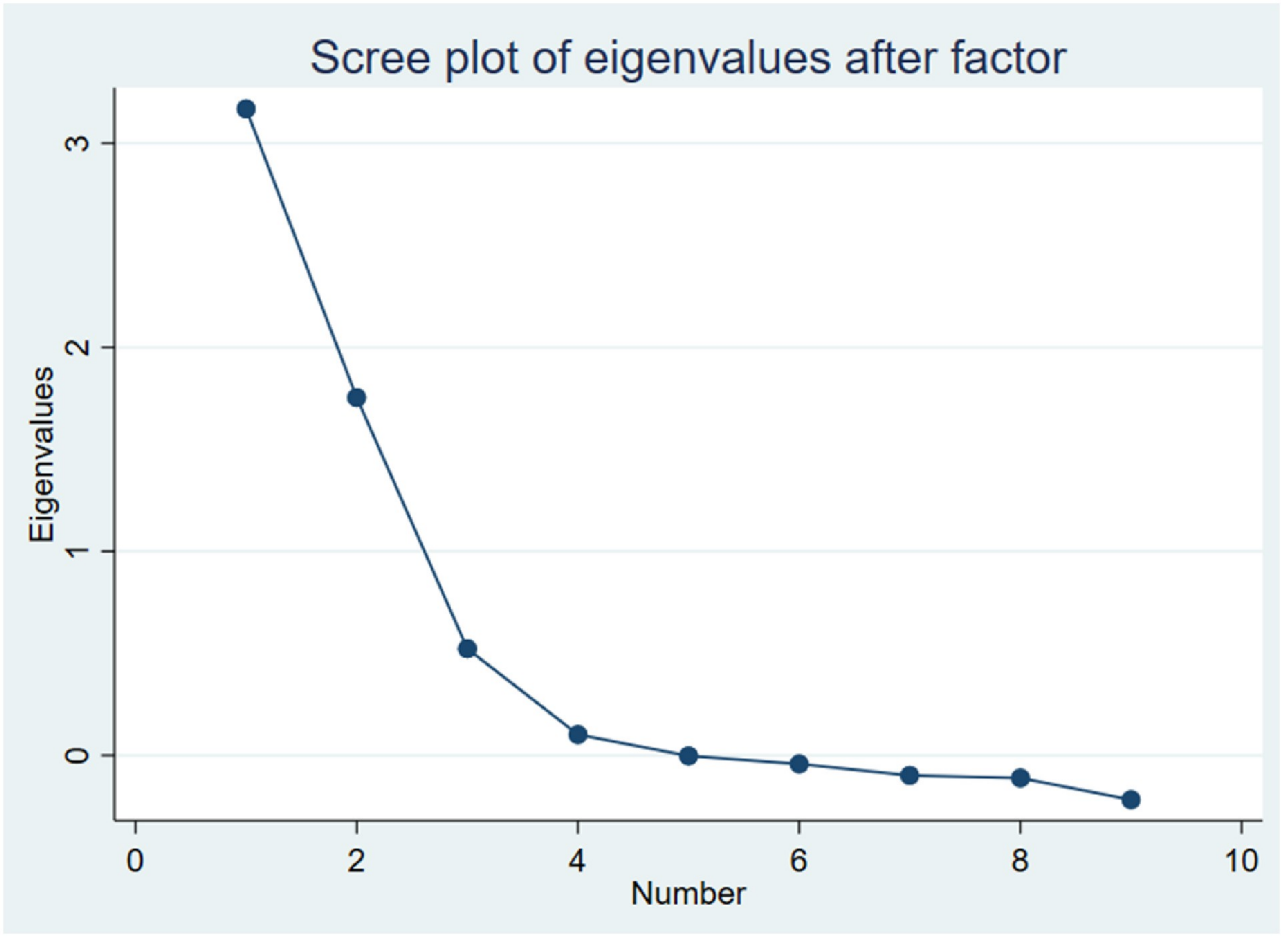
Scree plot of eigenvalues for the exploratory factor analysis.

Two items (age at initial onset of back pain, and presence of chronic back pain [Q1]) incorporated into one question on the screener were identified as potential screening questions in the baseline study and thus were excluded from the analysis. Preliminary analysis revealed that question 19 did not have sufficient factor loading (CITC < 0.3) and thus it was eliminated. Exploratory factor analysis was subsequently performed on the remaining nine questions using a principal component factor analysis with a varimax rotation. Results from this analysis (Table 5) generated a two-factor model to explain the latent variable. Additionally, the scree plot showed two factors above the elbow (Eigenvalues > 1), confirming that a two-factor solution is the best approach (Fig 2). Items that loaded highly on factor one included measures related to the impact of pain on daily life (Q3, Q9, Q11, Q13, and Q14); these were designated as “daily life functioning”. Items relating to the effect of pain on movement (Q5 and Q7) loaded on factor two and were designated as “pain better with movement”.

**Table 5:** Results of rotated factor loadings of exploratory factor analysis.

Variables	Factor 1 (pain impacts daily life functioning)	Factor 2 (pain better with movement)
Q3. Over the last 3 months, how often did your back feel stiff during the first two hours after waking?	0.77	
Q4. Over the last 3 months, how often have you experienced a decreased range of motion?	0.58	
Q5. Over the last 3 months, how often did your back pain get better with movement?		0.90
Q7. Over the last 3 months, how often did your back pain get better with movement within two hours after waking?		0.90
Q9. Over the last 3 months, how often did your back pain make it uncomfortable to sit for more than 2 hours?	0.60	
Q11. Over the last 3 months, how often do you avoid social activities because of your back pain?	0.69	
Q13. Over the last 3 months, how often did your back pain make it difficult to sleep?	0.80	
Q14. Over the last 3 months, how often did your back pain wake you up from sleep?	0.79	
Q15. Over the last 3 months, how often did you have pain in your hip, neck, heel, or elbow?	0.31	

*Blanks represent loading <0.3.

### Cronbach’s alpha

The instrument (including questions extracted from the exploratory factor analysis) was found to have satisfactory internal consistency with a Cronbach’s alpha of 0.81. The Cronbach’s alpha coefficient remained unchanged following the exclusion of two items (Q4 and Q15). Because two items (Q5 and Q7) that loaded on factor 2 (pain better with movement) were found to correlate highly with one another, we included only one of these two variables in the screener (Q5). Table 6 lists the six questions that were included in the final proposed screening tool.

**Table 6:** List of questions included in the final proposed screening tool.

Q3. Over the last 3 months, how often did your back feel stiff during the first two hours after waking?
Q5. Over the last 3 months, how often did your back pain get better with movement?
Q9. Over the last 3 months, how often did your back pain make it uncomfortable to sit for more than 2 hours?
Q11. Over the last 3 months, how often do you avoid social activities because of your back pain?
Q13. Over the last 3 months, how often did your back pain make it difficult to sleep?
Q14. Over the last 3 months, how often did your back pain wake you up from sleep?

### Content validity

Most of the 21 questions that were evaluated for content validity were considered by rheumatologists to be between “somewhat important” and “very important.” Three questions garnered mixed responses (Q8: Over the last 3 months, how often did your back pain get better with movement after waking? Q11: Over the last 3 months, how often did your back pain make it uncomfortable to sit for more than 2 hours? and Q21: Has anyone in your family had an auto immune disease?) and two questions were considered to be “insignificant/unimportant” (Q2: Over the last 3 months, how often did your back feel the same or worse? and Q10: Over the last 3 months, how often are you aware of your back pain when sitting for two hours or more?). The final six-item questionnaire (Table 6) was developed based upon feedback from both patients and the panel of rheumatologists to ensure that it incorporated the perspective of both groups.

## Discussion

Findings from the test-retest study indicate that our proposed screener is fairly stable. The test-retest reliabilities were acceptable, with eleven items having quadratic weighted Kappa values of ≥0.6, six items with values ≥0.5 and <0.6, and only 2 items with values <0.5. The exploratory factor analysis extracted two factors related to: 1) impact of pain on life and 2) impact of movement on pain. The internal consistency for questions evaluating the impact of pain on life was high, with a Cronbach’s alpha of 0.81. Six questions were identified for incorporation into a screening tool based on *a priori* psychometric properties. These final six questions have good test–retest reliability and high internal consistency.

Lower back pain is a common symptom and a frequent reason for seeking medical care in primary care settings in the US [34,35]. Differentiating common, relatively minor low back pain from the less common IBP which leads to an axSpA diagnosis is challenging [9,35]. Several strategies have been used previously to identify patients with axSpA [36–41]. These include testing for a single screening parameter (e.g., IBP) or a combination of parameters (e.g., HLA-B27 positivity, sacroiliitis on imaging, response to non-steroidal anti-inflammatory drugs, positive family history for SpA), performing whole body MRI, or applying ASAS classification criteria for axSpA [36–38,40,41]. However, questions related to a single parameter (e.g., IBP) showed low to no diagnostic value in primary care settings [37] and axSpA classification criteria were not developed for use as a screener [36,42]. In addition, Weisman et al, developed and validated a screening tool which combined laboratory test results and patient questionnaires to differentiate potential cases of AS from mechanical back pain [42]. However, the generalizability of this tool is a potential concern, since all of the patients included in their validation study had an established diagnosis of AS. Furthermore, the questionnaire may not include language reflective of the patient experiences.

Our prognostic screening tool improves upon these approaches. The proposed screening tool was informed by previous qualitative research and includes questions designed to test for a variety of axSpA symptoms [17]. Overall, we tested 19 items to capture multiple domains related to self-reported symptoms of axSpA, including IBP, stiffness, pain with movement, sleep, use of pain medications, other joint pain, and family history. For each domain, multiple questions were included that captured essentially the same underlying construct. Through a process of multiple iterations, only those items which scored well under domains that were most relevant to axSpA, according to published literature and our previous work [17,43–45], were included to develop a patient-friendly screener. For example, from the potential screening questions, three questions (Q5, Q6, Q7) related to movement were examined. The test-retest reliabilities for two (Q5 and Q7) of the three questions pertaining to movement had acceptable weighted kappa values (>0.6) and factor loadings (0.9), which ensured that ‘pain with movement’ was an important construct in patients with axSpA. Question 12 had low test-retest reliability (0.46), with both physicians and patients pointing out the ambiguity of the term “rest” and therefore was excluded. In addition, the family history question experienced mixed agreement from rheumatologists and in our previous study with cognitive interviews with mechanical back pain and diagnosed axSpA patients believed the question needed specific examples [17].

The primary goal of this study was to develop a patient-friendly screener that reflects patient experiences. Considering the complexity of the US healthcare system and the challenges associated with implementation of a screening tool in primary care settings [15,46], evidence to ensure its usefulness in routine primary care practice will be important. In our previous work, we demonstrated that the proposed screener possesses the necessary properties to serve as an effective screening tool: it is brief, understandable at a grade 8 reading level, and can be self-administered [17]. Further, it should help clinicians to decide which of the screened patients will benefit from prompt rheumatology referral for evaluation and treatment.

### Strengths and limitations

A notable strength of this study is that the study sample included individuals with any form of chronic back pain, all of whom were recruited from ResearchMatch. Thus, our results are more generalizable than those of previous studies that included only individuals with IBP. In addition, the proposed screening questions were informed by our previous work in which we explored issues related to patients’ diagnostic journeys using evidence-based qualitative research methodology. This helped us to understand and incorporate patient and physician perspectives [15,17,45]. Our study also has several limitations. Presently, the screening tool is available only in the English language. Considering that participation was voluntary and responses to the surveys were self-reported, there may be self-selection bias and recall bias. In addition, since the presence of chronic back pain was self-reported, the lack of physician confirmation of the diagnosis may have introduced further potential bias on the study. Since axSpA is known to manifest differently in men and women, a sex-stratified analysis would have been ideal and informative. However, our study was not sufficiently powered to perform such an analysis. Further research to refine and validate the screening tool in multiple clinical settings, including family medicine, rheumatology, orthopedic, and spine clinics, is warranted.

## Conclusions

Diagnostic delays in axSpA are significant as patients often suffer with symptoms for years before being diagnosed, thereby contributing to the economic burden of this disease to patients, their caregivers, and the healthcare system. Early recognition and awareness of axSpA symptoms in the primary care setting, primarily those of IBP, is paramount to reduce disease burden and improve quality of life in these patients. To be feasible for its implementation in routine clinical care, a screening tool must be brief and capture distinguishing features of axSpA. Further areas of exploration include the inclusion of gender and ethnic demographics. This study provides core information from which several versions of a screening tool for axSpA may be developed for validation in future studies. An evidence-based approach will be critical to demonstrate the validity and effectiveness of such a screening tool to support its widespread implementation in primary care.

## Supporting information

S1 File Baseline survey. (DOCX)

S2 File Follow-up survey. (DOCX)

S3 File Rheumatologist questionnaire. (DOCX)

## References

[1] Braun J, Sieper J. Ankylosing spondylitis. The Lancet. 2007;369(9570):1379–90. 10.1016/S0140-6736(07)60635-7 17448825

[2] Bandinelli F, Salvadorini G, Delle Sedie A, Riente L, Bombardieri S, Matucci-Cerinic M. Impact of gender, work, and clinical presentation on diagnostic delay in Italian patients with primary ankylosing spondylitis. Clinical rheumatology. 2016;35(2):473–8. doi: 10.1007/s10067-015-3005-z 26238665

[3] Spondilitis Association of America. Has Your Patient Had Inflammatory Back Pain for 3+ Months? Accessed October 27, 2020. https://www.spondylitis.org/For-Primary-Care-Physicians.

[4] Deodhar A, Mease PJ, Reveille JD, Curtis JR, Chen S, Malhotra K, et al. Frequency of axial spondyloarthritis diagnosis among patients seen by US rheumatologists for evaluation of chronic back pain. Arthritis & Rheumatology. 2016;68(7):1669–76. doi: 10.1002/art.39612 26816002 PMC5094500

[5] Sykes MP, Doll H, Sengupta R, Gaffney K. Delay to diagnosis in axial spondyloarthritis: are we improving in the UK? Rheumatology. 2015;54(12):2283–4. doi: 10.1093/rheumatology/kev288 26283680

[6] Waddell G, Burton AK. Occupational health guidelines for the management of low back pain at work: evidence review. Occupational medicine. 2001;51(2):124–35. doi: 10.1093/occmed/51.2.124 11307688

[7] Sieper J, Braun J, Rudwaleit M, Boonen A, Zink A. Ankylosing spondylitis: an overview. Annals of the rheumatic diseases. 2002;61(suppl 3):iii8–iii18. doi: 10.1136/ard.61.suppl_3.iii8 12381506 PMC1766729

[8] Mau W, Zeidler H, Mau R, Majewski A, Freyschmidt J, Stangel W, et al. Clinical features and prognosis of patients with possible ankylosing spondylitis. Results of a 10-year followup. The Journal of Rheumatology. 1988;15(7):1109. 3262757

[9] Danve A, Deodhar A. Axial spondyloarthritis in the USA: diagnostic challenges and missed opportunities. Clinical rheumatology. 2019;38(3):625–34. doi: 10.1007/s10067-018-4397-3 30588555

[10] Strand V, Singh JA. Patient burden of axial spondyloarthritis. Journal of Clinical Rheumatology. 2017;23(7):383. doi: 10.1097/RHU.0000000000000589 28937474 PMC5617559

[11] Barnett R, Ingram T, Sengupta R. Axial spondyloarthritis 10 years on: still looking for the lost tribe. Rheumatology. 2020;59(Supplement_4):iv25–iv37. doi: 10.1093/rheumatology/keaa472 33053196 PMC7566532

[12] van Onna M, Gorter S, van Meerendonk A, van Tubergen A. General practitioners’ perceptions of their ability to identify and refer patients with suspected axial spondyloarthritis: a qualitative study. The Journal of rheumatology. 2014;41(5):897–901. doi: 10.3899/jrheum.131293 24692524

[13] Magrey M, Yi E, Wolin D, Price M, Chirila C, Davenport E, et al., editors. Recognition of inflammatory back pain by US healthcare providers and barriers to specialist referral. ARTHRITIS & RHEUMATOLOGY; 2019: WILEY 111 RIVER ST, HOBOKEN 07030–5774, NJ USA.

[14] Jois R, Macgregor A, Gaffney K. Recognition of inflammatory back pain and ankylosing spondylitis in primary care. Rheumatology. 2008;47(9):1364–6. doi: 10.1093/rheumatology/ken224 18577550

[15] Lapane KL, Shridharmurthy D, Khan S, Lindstrom D, Beccia A, Yi E, et al. Primary care physician perspectives on screening for axial spondyloarthritis: A qualitative study. Plos one. 2021;16(5):e0252018. doi: 10.1371/journal.pone.0252018 34029339 PMC8143395

[16] Akkoc N, Khan MA. ASAS classification criteria for axial spondyloarthritis: time to modify. Clinical rheumatology. 2016;35(6):1415–23. doi: 10.1007/s10067-016-3261-6 27094940

[17] Divya Shridharmurthy* SK, Kate L. Lapane, Esther Yi, Jonathan Kay, Shao-Hsien Liu. Development of a screening tool to identify patients with axial spondyloarthritis: A cognitive interview study. BMC Health Services Research. 2021. 10.1007/s10067-022-06072-8 35059882

[18] Harris PA, Taylor R, Minor BL, Elliott V, Fernandez M, O’Neal L, et al. The REDCap consortium: Building an international community of software platform partners. Journal of biomedical informatics. 2019;95:103208. doi: 10.1016/j.jbi.2019.103208 31078660 PMC7254481

[19] Nemoto T, Beglar D, editors. Likert-scale questionnaires. JALT 2013 conference proceedings; 2014.

[20] Harris PA, Scott KW, Lebo L, Hassan N, Lighter C, Pulley J. ResearchMatch: a national registry to recruit volunteers for clinical research. Academic medicine: journal of the Association of American Medical Colleges. 2012;87(1):66. 22104055 10.1097/ACM.0b013e31823ab7d2 PMC3688834

[21] Landis JR, Koch GG. The measurement of observer agreement for categorical data. biometrics. 1977:159–74. 843571

[22] Cohen J. A coefficient of agreement for nominal scales. Educational and psychological measurement. 1960;20(1):37–46.

[23] Cohen J. Weighted kappa: nominal scale agreement provision for scaled disagreement or partial credit. Psychological bulletin. 1968;70(4):213. doi: 10.1037/h0026256 19673146

[24] Fleiss JL, Cohen J. The equivalence of weighted kappa and the intraclass correlation coefficient as measures of reliability. Educational and psychological measurement. 1973;33(3):613–9.

[25] McHugh ML. Interrater reliability: the kappa statistic. Biochemia medica: Biochemia medica. 2012;22(3):276–82. 23092060 PMC3900052

[26] Fleiss JL. Measuring nominal scale agreement among many raters. Psychol Bull. 1971;76(5):378–382. doi: 10.1037/h0031619

[27] Shrout PE, Fleiss JL. Intraclass correlations: uses in assessing rater reliability. Psychological bulletin. 1979;86(2):420. doi: 10.1037//0033-2909.86.2.420 18839484

[28] Nunnally JC. Psychometric theory 3E: Tata McGraw-hill education; 1994.

[29] Hair J. W C; Babin BJ; Anderson RE Multivariate data analysis. Upper Saddle River, NJ: Prentice Hall; 2010.

[30] Kaiser HF. The application of electronic computers to factor analysis. Educational and psychological measurement. 1960;20(1):141–51.

[31] Cattell RB. The Scree Test For The Number Of Factors. Multivariate Behav Res. 1966;1(2):245–276. doi: 10.1207/s15327906mbr0102_10 26828106

[32] Cronbach LJ. Coefficient alpha and the internal structure of tests. psychometrika. 1951;16(3):297–334.

[33] Tavakol M., & Dennick R. (2011). Making sense of Cronbach’s alpha. International journal of medical education, 2, 53–55. 10.5116/ijme.4dfb.8dfd 28029643 PMC4205511

[34] World Health Organization. WHO Background Paper 6.24 Low Back Pain. http://www.who.int/medicines/areas/priority_medicines/Ch6_24LBP.pdf?ua=1.

[35] Lawrence RC, Felson DT, Helmick CG, Arnold LM, Choi H, Deyo RA, et al. Estimates of the prevalence of arthritis and other rheumatic conditions in the United States: Part II. Arthritis & Rheumatism. 2008;58(1):26–35. doi: 10.1002/art.23176 18163497 PMC3266664

[36] Hermann J, Giessauf H, Schaffler G, Ofner P, Graninger W. Early spondyloarthritis: usefulness of clinical screening. Rheumatology. 2009;48(7):812–6. doi: 10.1093/rheumatology/kep119 19447774

[37] Braun A, Saracbasi E, Grifka J, Schnitker J, Braun J. Identifying patients with axial spondyloarthritis in primary care: how useful are items indicative of inflammatory back pain? Annals of the rheumatic diseases. 2011;70(10):1782–7. doi: 10.1136/ard.2011.151167 21821621 PMC3171105

[38] Braun J, Inman R. Clinical significance of inflammatory back pain for diagnosis and screening of patients with axial spondyloarthritis. Annals of the Rheumatic Diseases. 2010;69(7):1264–8. doi: 10.1136/ard.2010.130559 20566619

[39] Poddubnyy D, Vahldiek J, Spiller I, Buss B, Listing J, Rudwaleit M, et al. Evaluation of 2 screening strategies for early identification of patients with axial spondyloarthritis in primary care. The Journal of rheumatology. 2011;38(11):2452–60. doi: 10.3899/jrheum.110070 21921100

[40] Althoff C, Appel H, Rudwaleit M, Sieper J, Eshed I, Hamm B, et al. Whole-body MRI as a new screening tool for detecting axial and peripheral manifestations of spondyloarthritis. Annals of the rheumatic diseases. 2007;66(7):983–5. doi: 10.1136/ard.2007.069948 17576787 PMC1955100

[41] Poddubnyy D, van Tubergen A, Landewé R, Sieper J, van der Heijde D. Development of an ASAS-endorsed recommendation for the early referral of patients with a suspicion of axial spondyloarthritis. Annals of the rheumatic diseases. 2015;74(8):1483–7. doi: 10.1136/annrheumdis-2014-207151 25990288

[42] Weisman MH, Chen L, Clegg DO, Davis JC Jr, Dubois RW, Prete PE, et al. Development and validation of a case ascertainment tool for ankylosing spondylitis. Arthritis Care & Research: Official Journal of the American College of Rheumatology. 2010;62(1):19–27. doi: 10.1002/acr.20009 20191487

[43] Rojas-Vargas M, Munoz-Gomariz E, Escudero A, Font P, Zarco P, Almodovar R, et al. First signs and symptoms of spondyloarthritis—data from an inception cohort with a disease course of two years or less (REGISPONSER-Early). Rheumatology. 2009;48(4):404–9. doi: 10.1093/rheumatology/ken506 19208685 PMC2656634

[44] Otón T, Sastre C, Carmona L. The journey of the non-radiographic axial spondyloarthritis patient: the perspective of professionals and patients. Clinical Rheumatology. 2021;40(2):591–600. doi: 10.1007/s10067-020-05269-z 32632698

[45] Dube CE, Lapane KL, Ferrucci KA, Beccia AL, Khan SK, Yi E, et al. Personal Experiences with Diagnostic Delay Among Axial Spondyloarthritis Patients: A Qualitative Study. Rheumatology and Therapy. 2021:1–16. doi: 10.1007/s40744-021-00321-z 34059989 PMC8217406

[46] Lapane KL, Khan S, Shridharmurthy D, Beccia A, Dubé C, Yi E, et al. Primary care physician perspectives on barriers to diagnosing axial Spondyloarthritis: a qualitative study. BMC Family Practice. 2020;21(1):1–11. 32993510 10.1186/s12875-020-01274-y PMC7526414

