# Supplementary material for: Development and test–retest reliability of a screening tool for axial spondyloarthritis
Source: PLoS One. 2022 Jul 8;17(7):e0269494. doi: 10.1371/journal.pone.0269494 (PMC9269406; doi:10.1371/journal.pone.0269494)
Supplement: S1 File — (DOCX) [file pone.0269494.s001.docx]

Thank you for your interest in this research study!

We are in the process of developing a screening questionnaire to help identify patients who may be in need of further work-up for their back pain. The goal of the research project is to develop a non-invasive screening questionnaire to help reduce diagnostic delay for patients with certain types of back pain.

First, please answer these two questions to determine if you are eligible for this study.

1. Have you suffered from back pain for less than 3 months? If yes, STOP

□ YES 🡪 STOP “Thank you for your interest in our study. Unfortunately, you are not eligible for participation.”

□ NO🡪 continue to next question

1. Was there a specific incident you remember that caused your back pain (like an injury or accident)?

□ YES 🡪 STOP “Thank you for your interest in our study. Unfortunately, you are not eligible for participation.”

□ NO🡪 continue to next question

1. You are eligible to participate in this study! Before proceeding, please know that there is always the possibility of tampering from an outside source when using the internet for collecting information. While the confidentiality of your responses will be protected once the data are downloaded from the internet, there is always a possibility of hacking or other security breaches that could threaten the confidentiality of your responses. Please know that you are free to decide not to answer any question.

Would like to participate in this study?

□ No 🡪 STOP “Thank you for your time.”

□ Yes🡪 continue to next question

Thank you for agreeing to participate in this research project.

1. Over the last 3 months, how often have you suffered from back pain without a known cause?

□ Nearly every day □ More than half the days □ Several days □ Not at all □ Prefer not to answer

1. Over the last 3 months, how often did your back feel the same or worse?

□ Nearly every day □ More than half the days □ Several days □ Not at all □ Prefer not to answer

1. Over the last 3 months, how often did your back feel stiff during the first two hours after waking?

□ Nearly every day □ More than half the days □ Several days □ Not at all □ Prefer not to answer

1. Over the last 3 months, how often have you experienced a decreased range of motion?

□ Nearly every day □ More than half the days □ Several days □ Not at all □ Prefer not to answer

1. Over the last 3 months, how often did your back pain get better with movement?

□ Nearly every day □ More than half the days □ Several days □ Not at all □ Prefer not to answer

1. Over the last 3 months, how often did your back pain get better with movement after waking?

□ Nearly every day □ More than half the days □ Several days □ Not at all □ Prefer not to answer

1. Over the last 3 months, how often did your back pain get better with movement within two hours after waking?

□ Nearly every day □ More than half the days □ Several days □ Not at all □ Prefer not to answer

1. Over the last 3 months, how often have you been unable to sit still for more than two hours because of your back pain?

□ Nearly every day □ More than half the days □ Several days □ Not at all □ Prefer not to answer

1. Over the last 3 months, how often did your back pain make it uncomfortable to sit for more than 2 hours?

□ Nearly every day □ More than half the days □ Several days □ Not at all □ Prefer not to answer

1. Over the last 3 months, how often are you aware of your back pain when sitting for two hours or more?

□ Nearly every day □ More than half the days □ Several days □ Not at all □ Prefer not to answer

1. Over the last 3 months, how often do you avoid social activities because of your back pain?

□ Nearly every day □ More than half the days □ Several days □ Not at all □ Prefer not to answer

1. Over the last 3 months, how often did your back pain ease with rest?

□ Nearly every day □ More than half the days □ Several days □ Not at all □ Prefer not to answer

1. Over the last 3 months, how often did your back pain make it difficult to sleep?

□ Nearly every day □ More than half the days □ Several days □ Not at all □ Prefer not to answer

1. Over the last 3 months, how often did your back pain wake you up from sleep?

□ Nearly every day □ More than half the days □ Several days □ Not at all □ Prefer not to answer

1. Over the last 3 months, how often did you have pain in your hip, neck, heel, or elbow?

□ Nearly every day □ More than half the days □ Several days □ Not at all □ Prefer not to answer

1. Over the last 3 months, how often have you experienced alternating pain in your hips or buttocks?

□ Nearly every day □ More than half the days □ Several days □ Not at all □ Prefer not to answer

1. Over the last 3 months, how often have you experienced improvement in your back pain after taking non-steroidal anti-inflammatory medications (like naproxen, ibuprofen, etc)?

□ Nearly every day □ More than half the days □ Several days □ Not at all □ Prefer not to answer

1. Has a doctor ever told you that you have an autoimmune disease like iritis, Crohn’s disease, or psoriasis? (Yes/No)

□ YES

□ NO

1. Has anyone in your family had an auto immune disease? (Yes/No)

□ YES

□ NO

1. Are you a

□ Man □ Woman □ Non-binary □ Prefer not to answer

1. What is your age? _____ (If you prefer not to answer, please enter 999)
2. At what age did your back pain begin? _____ (If you prefer not to answer, please enter 999)
3. Are you Hispanic or Latino?

□ YES

□ NO

1. Do you consider yourself to be:

□ White □ Black or African American □ Asian □ Hawaiian or Other Pacific Islander

□ Native American Indian or Alaskan Native

□ Multiracial □ Prefer not to answer

1. What is your highest level of education

□ Less than high school □ Completed High School or GED □ Some college or technical school

□ Completed College □ Graduate school □ Prefer not to answer

Thank you for your time! As a reminder, we will email you in 14 days to complete the second survey.

Please click on the link below to submit. You will be directed to another data base where you will be asked to enter your name and an email address where we will forward the link to your gift card.

In REDCap - we will have the person enter their name and email address where they would like us to send the cash card link. *** There will be a separate from their responses here.
