# Supplementary material for: Development and test–retest reliability of a screening tool for axial spondyloarthritis
Source: PLoS One. 2022 Jul 8;17(7):e0269494. doi: 10.1371/journal.pone.0269494 (PMC9269406; doi:10.1371/journal.pone.0269494)
Supplement: S3 File — (DOCX) [file pone.0269494.s003.docx]

Thank you for agreeing to participate in this preparatory work for research.

Before proceeding, please know that there is always the possibility of tampering from an outside source when using the internet for collecting information. While the confidentiality of your responses will be protected once the data are downloaded from the internet, there is always a possibility of hacking or other security breaches that could threaten the confidentiality of your responses. Please know that you are free to decide not to answer any question.”

Would like to continue?

□ No 🡪 STOP “Thank you for your time.”

□ Yes🡪 continue to directions

**Directions:**

We are in the process of developing a screening questionnaire to help identify patients who may be in need of further work-up for axial spondyloarthritis. The goal of the research project is to develop a non-invasive screening questionnaire to help reduce diagnostic delay for patients with axial spondyloarthritis. We are preparing to conduct a test-retest reliability study with patients with chronic back pain. To prepare for this research project, we would like input from rheumatologists. Based on previous qualitative research with primary care providers and patients, we have identified a series of potential questions for the axial spondyloarthritis screening tool. Unless otherwise indicated, the response set patient’s will be provided are: Nearly every day, More than half the days, Several days, Not at all.

We would appreciate it if you would rate the following conceptual areas as to how important YOU BELIEVE each item is for identifying patients who may have undiagnosed axial spondyloarthritis. We would also appreciate any comments you choose to provide about the items.

**Thank you.**

1. Have you suffered from back pain for less than 3 months? If yes, STOP

□ Not important □ Somewhat important □ Very important (must select one of the 3 options)

1b) Comments

__________________________________________ (open ended text box)

1. Was there a specific incident you remember that caused your back pain (like an injury or accident)? If yes, STOP

□ Not important □ Somewhat important □ Very important (must select one of the 3 options)

2b) Comments

__________________________________________ (open ended text box)

1. Over the last 3 months, how often have you suffered from back pain without a known cause?

□ Not important □ Somewhat important □ Very important (must select one of the 3 options)

3b) Comments

__________________________________________ (open ended text box)

1. Over the last 3 months, how often did your back feel the same or worse?

□ Not important □ Somewhat important □ Very important (must select one of the 3 options)

4b) Comments

__________________________________________ (open ended text box)

1. Over the last 3 months, how often did your back feel stiff during the first two hours after waking?

□ Not important □ Somewhat important □ Very important (must select one of the 3 options)

5b) Comments

__________________________________________ (open ended text box)

1. Over the last 3 months, how often have you experienced a decreased range of motion?

□ Not important □ Somewhat important □ Very important (must select one of the 3 options)

6b) Comments

__________________________________________ (open ended text box)

1. Over the last 3 months, how often did your back pain get better with movement?

□ Not important □ Somewhat important □ Very important (must select one of the 3 options)

7b) Comments

__________________________________________ (open ended text box)

1. Over the last 3 months, how often did your back pain get better with movement after waking?

□ Not important □ Somewhat important □ Very important (must select one of the 3 options)

8b) Comments

__________________________________________ (open ended text box)

1. Over the last 3 months, how often did your back pain get better with movement within two hours after waking?

□ Not important □ Somewhat important □ Very important (must select one of the 3 options)

9b) Comments

__________________________________________ (open ended text box)

1. Over the last 3 months, how often have you been unable to sit still for more than two hours because of your back pain?

□ Not important □ Somewhat important □ Very important (must select one of the 3 options)

10b) Comments

__________________________________________ (open ended text box)

1. Over the last 3 months, how often did your back pain make it uncomfortable to sit for more than 2 hours?

□ Not important □ Somewhat important □ Very important (must select one of the 3 options)

11b) Comments

__________________________________________ (open ended text box)

1. Over the last 3 months, how often are you aware of your back pain when sitting for two hours or more?

□ Not important □ Somewhat important □ Very important (must select one of the 3 options)

12b) Comments

__________________________________________ (open ended text box)

1. Over the last 3 months, how often do you avoid social activities because of your back pain?

□ Not important □ Somewhat important □ Very important (must select one of the 3 options)

13b) Comments

__________________________________________ (open ended text box)

1. Over the last 3 months, how often did your back pain ease with rest?

□ Not important □ Somewhat important □ Very important (must select one of the 3 options)

14b) Comments

__________________________________________ (open ended text box)

1. Over the last 3 months, how often did your back pain make it difficult to sleep?

□ Not important □ Somewhat important □ Very important (must select one of the 3 options)

15b) Comments

__________________________________________ (open ended text box)

1. Over the last 3 months, how often did your back pain wake you up from sleep?

□ Not important □ Somewhat important □ Very important (must select one of the 3 options)

16b) Comments

__________________________________________ (open ended text box)

1. Over the last 3 months, how often did you have pain in your hip, neck, heel, or elbow?

□ Not important □ Somewhat important □ Very important (must select one of the 3 options)

17b) Comments

__________________________________________ (open ended text box)

1. Over the last 3 months, how often have you experienced alternating pain in your hips or buttocks?

□ Not important □ Somewhat important □ Very important (must select one of the 3 options)

18b) Comments

__________________________________________ (open ended text box)

1. Over the last 3 months, how often have you experienced improvement in your back pain after taking non-steroidal anti-inflammatory medications (like naproxen, ibuprofen, etc)?

□ Not important □ Somewhat important □ Very important (must select one of the 3 options)

19b) Comments

__________________________________________ (open ended text box)

1. Has a doctor ever told you that you have an autoimmune disease like iritis, Crohn’s disease, or psoriasis? (Yes/No)

□ Not important □ Somewhat important □ Very important (must select one of the 3 options)

20b) Comments

__________________________________________ (open ended text box)

1. Has anyone in your family had an auto immune disease? (Yes/No)

□ Not important □ Somewhat important □ Very important (must select one of the 3 options)

21b) Comments

__________________________________________ (open ended text box)

Thank you for your time.

Please click on the link below to submit. You will be directed to another form to enter your name and an email address where we will forward the link to your gift card.

In REDCap – we will have the physician participant enter their name and email address where they would like us to send the cash card link. *** There will be a separate sheet from their responses here.
